# BridgeDb app: unifying identifier mapping services for Cytoscape

**DOI:** 10.12688/f1000research.4521.1

**Published:** 2014-07-01

**Authors:** Jianjiong Gao, Chao Zhang, Martijn van Iersel, Li Zhang, Dong Xu, Nikolaus Schultz, Alexander R. Pico

**Affiliations:** 1 Kravis Center for Molecular Oncology, Memorial Sloan Kettering Cancer Center, New York, NY 10065, USA; 2 Institute for Computational Biomedicine, Weill Cornell Medical College, New York, NY 10065, USA; 3 General Bioinformatics, Reading, Birkshire, RG4 7RT, UK; 4 School of Computer Science and Engineering, Changchun University of Technology, Changchun, 130012, China; 5 Department of Computer Science,C.S. Bond Life Sciences Center, University of Missouri, Columbia, MO 65211, USA; 6 Department of Epidemiology and Biostatistics, Memorial Sloan Kettering Cancer Center, New York, NY 10065, USA; 7 Gladstone Institutes, San Francisco, CA 94158, USA

## Abstract

The BridgeDb app for Cytoscape allows users to map and annotate identifiers of genes, proteins and metabolites in the context of biological networks. The app greatly simplifies the identifier mapping process in Cytoscape by providing a unified interface to different mapping resources and services. The app also provides a programming interface via Cytoscape Commands that can be utilized for identifier mapping by other Cytoscape apps. In this article we provide a technical guide to the BridgeDb app for mapping identifiers in Cytoscape.

## Introduction

Cytoscape ^1^ is a powerful network visualization tool and platform for data integration and analysis. However, identifier mapping remains a challenge when working with biological data from different sources. We developed the BridgeDb app for Cytoscape to provide utilities for mapping and annotating identifiers in the network context. Built on the BridgeDb open-source framework for identifier mapping ^2^, the BridgeDb app provides a graphical user interface (GUI) for users and a command interface for other Cytoscape apps to perform identifier mapping in Cytoscape networks. As a result, BridgeDb enables or simplifies the processes of identifier translation, biological entity unification, and functional annotation.

## Implementation

The BridgeDb identifier mapping framework ( http://bridgedb.org/) ^2^ was designed to provide standardized access to gene, protein and metabolite identifier mapping services such as Ensembl BioMart ^3^, Synergizer ^4^, PICR ^5^ and BridgeDb web services and BridgeDb database files. By creating a Java-based abstract layer, BridgeDb enables bioinformatics applications to connect to different mapping resources through the same interface, which greatly alleviates the burden of exploring, maintaining and switching between resources.

**Figure 1.**
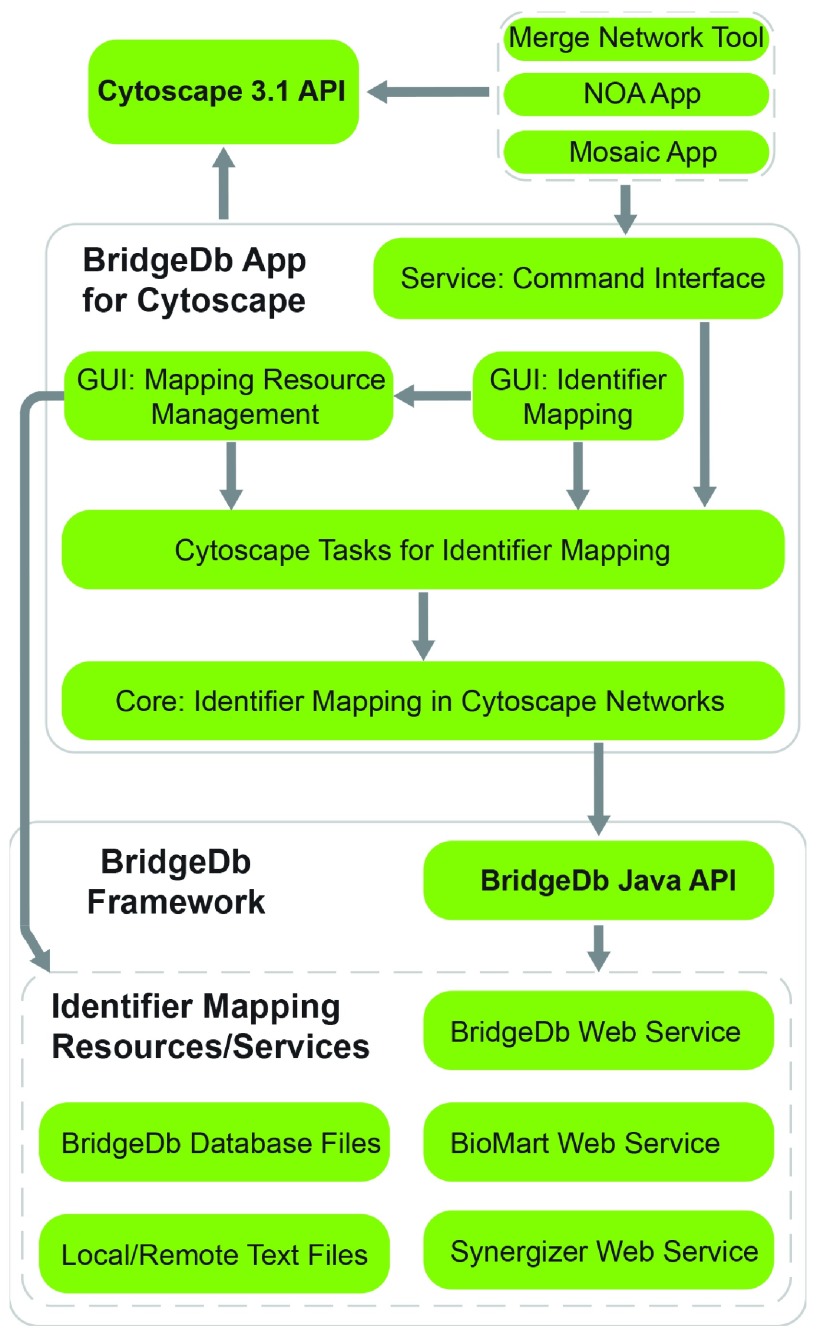
BridgeDb app implementation.

Built upon BridgeDb framework and API, the BridgeDb app for Cytoscape can be used to connect to different mapping resources and map identifiers in Cytoscape netwoks. The BridgeDb app was implemented based on the Cytoscape 3 API. Its predecessor was the CyThesarus plugin for Cytoscape 2. To take advantage of the new OSGi based architecture in Cytoscape 3 and its clearly defined and simplified API, we have rewritten the CyThesaurus plugin into an OSGi bundle app. Figure 1 illustrates the implementation details. The identifier mapping API of BridgeDb framework was wrapped by Cytoscape *Task* and *TaskFactory* API, which provide identifier mapping utilities to users through graphical user interfaces for managing mapping resources and performing identifier mapping. The BridgeDb app Tasks were also registered to Cytoscape as command services allowing other apps, such as Mosaic ^6^ and NOA ^7^ apps, and the Merge Network tool, to take advantage of BridgeDb app’s identifier mapping capacities.

## Results

### BridgeDb app user interface

The BridgeDb app provides an intuitive GUI to perform identifier mapping in Cytoscape networks, consisting of two interactive dialogs for resource management and identifier mapping, respectively, which can be opened via the menu item Apps/BridgeDb.

The resource management dialog allows user to add, remove, and select or deselect mapping resources. Once the resources are configured, they will be saved in a global property file and therefore shared among different Cytoscape sessions. Currently BridgeDb app supports local and remote delimited text files, BridgeDb database files, BridgeDb web service, BioMart web service, and Synergizer web service. Web services are easy to access and up to date and therefore are preferable for annotating small to medium-size networks (less than 1,000 nodes). It is highly recommended to download and use the BridgeDb database files for mapping identifiers in large networks in order to save time. Local delimited text files are useful when mapping between non-standard or customized identifiers. To avoid ambiguity, it is recommended to select only one resource unless multiple resources are believed to be complementary to each other. Particularly, resources for different species should not be selected at the same time. Database or text files are also preferable when reproducibility is essential. We will update the app to support more mapping resources as BridgeDb API keeps being developed.

The identifier mapping dialog is the main interface for mapping identifiers stored in the node table in the selected Cytoscape network. The user needs to choose the source identifier types, columns in the node table that contain the source identifiers, target identifier types, and columns to save the target identifiers. When mapping, all specified source identifier types in all selected resources will be queried for matching identifiers. If one source identifier can be mapped onto multiple target identifiers, all target identifiers can be saved as a list in the node table. If a target column exists in the node table, all values in the column will be overwritten with the target identifiers; otherwise, a new column will be created and filled with the target identifiers. Besides a set of supported identifier types (e.g., Entrez Gene ID and UniProt accession), a mapping resource such as BridgeDb database may also have a set of supported attributes (e.g., gene symbol and description).

### BridgeDb app command interface

The BridgeDb app also provides a set of Cytoscape commands, which can be used by scripting or by other Cytoscape apps (client apps) to take advantage of the identifier mapping capacities provided by BridgeDb app. Table 1 provides a selected list of commands supported by BridgeDb. Every command has an optional argument ‘appName’ in order to allow every client app to have its own set of identifier mapping resources. Morris *et al.* ( **setsApp** ^8^, *published together in the Cytoscape App collection* ^9^) provided an example of how to use commands in client apps.

**Table 1.** Selected BridgeDb app commands. Arguments with asterisks (*) are required.

Command	Arguments	Description
bridgedb resource add	**classPath**= IDMapper class path* **connString**= BridgeDb connection string* **displayName**= *display name* **appName**= *name of client Cytoscape app*	Add a mapping resource. appName is the name of the client Cytoscape app that is using this command.
bridgedb resource remove	**connString**= BridgeDb connection string* **appName**= *name of Cytoscape app*	Remove a mapping resource.
bridgedb resource select	**connString**= BridgeDb connection string* **select**= *true or false* **appName**= *name of client Cytoscape app*	Select or deselect an mapping resource.
bridgedb id mapping	**network**= Cytoscape network name* **sourceColumn**= source column in node table* **targetColumn**= source ID type* **sourceIdType**= target column in node table* **targetIdType**= target ID type* **appName**= *name of client Cytoscape app*	Map identifiers from a column in node table to another in a Cytoscape network.
bridgedb resource config dialog	**appName**= *name of client Cytoscape app*	Open the resource management/ configuration dialog.
**appName**= *name of client Cytoscape app*	bridgedb main dialog	Open the main BridgeDb app dialog.

### Example use cases

**Figure 2.**
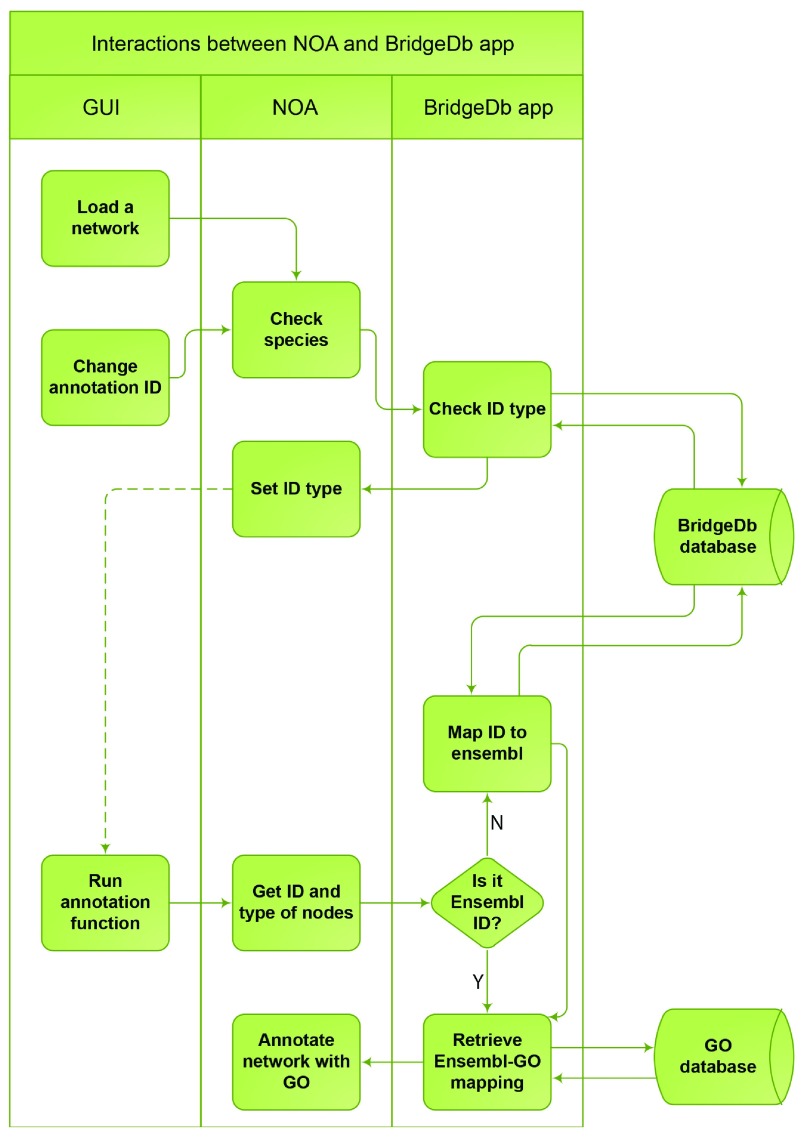
NOA app utilizing BridgeDb app services for gene ontology annotation.

Use cases of BridgeDb app include identifier translation, biological entity unification, and functional annotation in Cytoscape networks. Box 1 provides an example of how to use BridgeDb app to facilitate annotation and integration of networks from public databases. In particular, this example shows the steps to generate a *TP53* interaction network by merging networks from different sources using BridgeDb app and Merge Network tool. Figure 2 illustrates the process of the Cytoscape app NOA ^7^ annotating Cytoscape networks with Gene Ontology by utilizing BridgeDb app’s command services.

Box 1. **Example: Using BridgeDb app for merging TP53 interaction networks from different sources**

1.   Install and start Cytoscape 3.1.0 or above
2.   Select ***File > Import > Network > Public Databases. . .***
3.   Enter search condition ***TP53 AND human***, press ***Search***, select database ***IntAct***, and click ***Import***
4.   After importing, click ***No*** when asking about whether to manually merge networks, and close the import dialog
5.   If needed, install BridgeDb from ***Apps > App Manager***
6.   Select ***File > Import > Network > URL. . . > Example:Human Protein-Protein: Rual *et al.* . . . Nature 2005***
7.   Use default when asking about setting of the new network
8.   Select ***Apps > BridgeDb > Manager ID Mapping Resources***
9.   Click ***Databases***, Select database type ***.bridge***
10.   Click ***Download***, download ***Hs_Derby_[date].zip***, and unzip the file
11.   In Cytoscape, select the unzipped .bridge file
12.   Review supported identifier types and click ***Close***
13.   Select network rual.sif in Network panel
14.   Select ***Apps > BridgeDb > Map Identifiers***
15.   Select ***name*** as the ***Source Column in Node Table***
16.   Select ***Entrez Gene as Source ID Type(s)***
17.   Select ***Uniprot/TrEMBL*** as ***Target ID Type***
18.   Click ***Insert*** in the ***destination ID types*** table
19.   Select ***Attribute: Symbol*** as the second ***Target ID Type***
20.   Click ***OK***, wait, then click ***No*** to close
21.   Search for ***TP53*** in the Cytoscape search box
22.   Select ***Select > Nodes > First Neighbors of Selected Nodes > Undirected***
23.   Select ***File > New > Network > From selected nodes, all edges***
24.   Select ***Tool > Merge > Networks. . .***
25.   Select the network from ***IntAct*** and ***rual.sif(1)***
26.   Click ***Advanced Network Merge***
27.   Select ***Matching columns: uniprotkb_accession*** for ***IntAct***, and ***Uniprot/TrEMBL*** for ***rual.sif(1)***
28.   Click ***Merge*** to get a TP53 interaction network merged from the two sources

## Conclusions

In this paper, we presented the BridgeDb app for Cytoscape. By providing a unified interface to various mapping resources, BridgeDb app enables identifier mapping in the network context and therefore greatly eases the process of data integration in Cytoscape.

### Software availability

Software available from: http://apps.cytoscape.org/apps/bridgedb

Latest source code: https://github.com/jjgao/bridgedb.cytoscape

Source code as at the time of publication: https://github.com/F1000Research/bridgedb.cytoscape

Archived source code as at the time of publication: http://www.dx.doi.org/10.5281/zenodo.10465 ^10^

License: Lesser GNU Public License 2.1: https://www.gnu.org/licenses/old-licenses/lgpl-2.1.html
