# Supplementary material for: BridgeDb app: unifying identifier mapping services for Cytoscape
Source: F1000Res. 2014 Jul 1;3:148. [Version 1] doi: 10.12688/f1000research.4521.1 (PMC4111116; doi:10.12688/f1000research.4521.1)
Supplement: Peer review history

# Peer review: Referee response for version 1

**DOI:** 10.5256/f1000research.4835.r5522

**Published:** 2014-07-22

**Authors:** Olivier Elemento

**Affiliations:** 1 Institute for Computational Biomedicine, Weill Cornell Medical College, New York, NY, USA

The paper by Gao *et. al*. describes a Cytoscape app/plugin for cross-database mapping of gene, protein and metabolite identifiers in biological networks. For example, the app can map EntrezGene identifiers to UniProt identifiers for all genes or selected genes in a Cytoscape network. This is an important and useful application that among other application enables the merging of networks obtained from different sources (as shown in demonstration). The app is reasonably user-friendly and intuitive. Most importantly, it does not require any programming knowledge and is accessible via a GUI. I appreciate that it either uses local downloaded files for mapping (for heavy usage) or BridgeDb web services (for smaller networks). The demo in the paper worked flawlessly on my MacBook Air. 

Potential future improvements may include ortholog mapping e.g. from mouse to human to enable cross-species network analyses.

I have read this submission. I believe that I have an appropriate level of expertise to confirm that it is of an acceptable scientific standard.

# Peer review: Referee response for version 1

**DOI:** 10.5256/f1000research.4835.r5304

**Published:** 2014-07-10

**Authors:** Benno Schwikowski

**Affiliations:** 1 Systems Biology Lab, Institut Pasteur, Paris, France

BridgeDB app provides Cytoscape users and app developers with powerful functionality to address the ID mapping problem in integrative data analysis. App user and command interfaces are well-documented; the workflow is functional and recommended for anyone wishing to learn how to use the app.As a minor suggestion, it would be reassuring to new users if they were able to validate the result of the workflow example in some way.This is a very useful extension of Cytoscape functionality with broad appeal.

I have read this submission. I believe that I have an appropriate level of expertise to confirm that it is of an acceptable scientific standard.
